# Editorial: Bacteriophages Isolation From the Environment and Their Antimicrobial Therapeutic Potential, Volume 2

**DOI:** 10.3389/fmicb.2022.847176

**Published:** 2022-01-26

**Authors:** Krishna Mohan Poluri, Robert Czajkowski

**Affiliations:** ^1^Department of Biosciences and Bioengineering and Centre for Nanotechnology, Indian Institute of Technology Roorkee, Roorkee, India; ^2^Laboratory of Biologically Active Compounds, Intercollegiate Faculty of Biotechnology, University of Gdansk and Medical University of Gdansk, University of Gdansk, Gdansk, Poland

**Keywords:** bacteriophage, phage therapy, multidrug-resistant bacteria, environmental phages, phage isolation, aquaculture

The emergence and spread of antibiotic-resistant bacteria was one of the leading global concerns of the last century (Zaman et al., 2017; Chokshi et al., 2019). Today, at the beginning of the twenty-first century, bacteria still hold accountability for preponderant infectious pathogenesis. In the war against them, nature has equipped us with bacterial viruses (named bacteriophages or phages) that dwell on bacteria and can readily kill them (Chibani-Chennoufi et al., 2004). With the advancements in science and medicine, humankind is thriving toward using, manipulating, and repurposing the bacteriophages as effective antibacterials (Burrowes et al., 2011; Chan et al., 2013).

However, the idea to use bacteriophages to combat bacterial infections is not entirely new (Wittebole et al., 2014). The first successful trials involving bacterial viruses against human and animal bacterial pathogens were done just after their initial discovery at the beginning of the twentieth century (Campbell, 2010). Unfortunately, these trials and treatments were virtually stopped when penicillin was successfully commercialized as the first antimicrobial (Gaynes, 2017).

The twenty-first century seems to be the opening of the second golden age of bacteriophages with the increasing number of new scientific publications on the topic as well as the expanding societal understanding and approval for the use of (alive) bacterial viruses to fight bacterial infections in medicine, veterinary, food microbiology and agriculture applications. This global rediscovery of bacteriophages is also reflected in this Research Topic.

One of the keystones to taking advantage of bacteriophages is identifying and thoroughly investigating them. The current thematic issue encloses several reports of isolation and characterization of lytic bacterial viruses to be used in medicine and veterinary. Khan et al. isolated an *Escherichia coli* C (phi x174 host) lytic phage named MSK and have exhaustively studied the genome of this novel phage. Authors have precisely distinguished 73 open reading frames (ORFs) having diverse functionalities, in which 46 of them showed marked similarity with the ORFs previously reported in Rtp group of bacteriophages. Belonging to the family of *Drexlerviridae*, MSK has exhibited potent lytic action against multidrug-resistant (MDR) *E. coli* and other pathogenic *E. coli, Pseudomonas syringae*, and *Salmonella anatum* strains. On similar lines, Xu et al. isolated another lytic bacteriophage from *Shigella flexneri*, named vB_ShiP-A7. This new member of *Podoviridae* rendered cogent antimicrobial effect against MDR strains of *Shigella flexneri* and *E. coli*, as evidenced from the *in vivo* murine based studies. A new virulent bacteriophage of the *Myoviridae* family vB_EcoM_swi3 (swi3) from swine feces has been isolated and characterized by Sui et al. This bacteriophage is effective against infections caused by *Salmonella enteritidis* and pathogenic *E. coli*, as evidenced from both *in vitro* and *in vivo* studies. Gorodnichev et al. isolated and characterized three virulent phages: Dep622, vB_KpnM_Seu621, and KpS8, possessing narrow specificity toward MDR *Klebsiella pneumoniae* with K23 capsule type. From the analyzed phages, Dep622 specifically demonstrated polysaccharide depolymerase activity and successfully protected *Galleria mellonella* larvae infected with MDR *K. pneumoniae* strain.

Overuse and misuse of antibiotics may frequently lead to drug resistance in bacterial strains, and the common antibiotics are at the edge of failing. In such a scenario, bacteriophages may provide an attractive non-antibiotic approach to control pathogenic bacteria. Kortright et al. delineated the possibility of acquired cross-resistance in *E. coli* using two potential antibacterial agents- phage T6, phage U115 and an antibiotic albicidin. The study established that independent, selective resistance to any of these three antibacterial agents provided resistance to the other two. Whole-genome and targeted sequencing analysis of 29 samples showed that they all possess Tsx porin as a common point of interaction. In expounding the antimicrobial activity of bacteriophages, Yang et al. affirmed the efficiency of the KPP10 phage in treating *Pseudomonas aeruginosa* strain D4-induced pneumonia mouse models when administered intranasally. The model showed a significantly lower level of immunological indicators [TNFα, IL-1β, and IFN-γ] of infection without any lysis induced endotoxic shock.

Apart from humans, livestock and aquaculture are highly susceptible to bacterial infections and may act as symptomatic or asymptomatic carriers compromising human health. Most of these bacteria have become MDR due to extensive misuse of antibiotics in the feedstocks (Martin et al., 2015). It is believed that, at least to some extent and in some applications, antibiotics may be replaced by environmental-friendly alternatives: bacteriophages. Pelyuntha et al. has reported isolation and application of phage cocktail showing potential antimicrobial activity against *Salmonella* spp., sampled from broiler farms in Thailand. The cocktail derived from the three phages displaying the highest lytic ability was documented to markedly reduce the growth of *Salmonella* sp. collected from different sources over the country without any specific drug resistance pattern. Xie et al. sampled *Salmonella* sp. from beef hide and soil to analyze the antibacterial efficiency of four previously reported genetically diverse groups of bacteriophages against the tested bacterial strains. The individual phages showed comparable activity to the cocktail titer in liquid culture without significant phage-resistance activity. Tetens et al. reported the isolation and morphological characterization of 11 *Siphoviridae*-like phages along with three strains of their host, *Staphylococcus hyicus*, causing exudative epidermitis (EE) infection in piglet farms over Germany. Genome sequencing of the isolates identified a novel virulent phage, PITT-1 (PMBT8) and a novel temperate phage, PITT-5 (PMBT9). Further gene sequencing studies of the sampled host-bacterial strains revealed two toxin-encoding genes *exhA* and *exhC*, among which *exhC*-positive *S. hyicus* strains were found to be very mildly lysed by the majority of the lytic phages. Li et al. have isolated a novel bacteriophage BUCT549 infecting *Vibrio alginolyticus* from a seafood market sewage in China. *V. alginolyticus* is reported to cause food poisoning and septicemia in humans. Phylogenetic and transmission electron microscopic studies of BUCT549 reveal its kin association to the viral family *Siphoviridae*. Donati et al. investigated the efficacy of FpV4 and FPSV-D22, a dual-component phage mixture in controlling *Flavobacterium psychrophilum* infection triggered mortality of rainbow trout fry fish using oral, bath and injection-based administration approaches. This study documented the efficacy of the phage administration pathway in treating rainbow trout fry syndrome and endorsed the oral administration of the viral cocktail.

We believe that replacing antibiotics with bacteriophages in the various applications to prevent and treat bacterial infections in humans and animals will continue in the twenty-first century. In addition, with the use of genetically modified viruses, it will be possible to develop solutions to use them in personalized medicine.

Finally, we are sincerely thankful to all the authors for contributing to this Research Topic. We also would like to acknowledge the toiling and admirable work of the reviewers and their critical assessments of the reviewed manuscripts.

## Author Contributions

Both authors listed have made a substantial, direct, and intellectual contribution to the work and approved it for publication.

## Conflict of Interest

The authors declare that the research was conducted in the absence of any commercial or financial relationships that could be construed as a potential conflict of interest.

## Publisher's Note

All claims expressed in this article are solely those of the authors and do not necessarily represent those of their affiliated organizations, or those of the publisher, the editors and the reviewers. Any product that may be evaluated in this article, or claim that may be made by its manufacturer, is not guaranteed or endorsed by the publisher.
